# Enhancing the efficiency of achieving optical transparency in live animals using absorbing molecules

**DOI:** 10.1117/1.JBO.31.5.054702

**Published:** 2026-02-13

**Authors:** Ting Sun, Jing Su, Yanjie Zhao, Xin Tie

**Affiliations:** a West China Hospital, Sichuan University, Department of Critical Care Medicine, Chengdu, China; b West China Hospital, Sichuan University, Department of Neurosurgery, Chengdu, China; c West China Hospital, Sichuan University, Precision Medicine Translational Research Center, Chengdu, China

**Keywords:** tissue clearing, optical transparency, tartrazine, 4-aminoantipyrine, live animals, mouse imaging

## Abstract

**Significance:**

*In vivo* optical imaging is crucial for studying disease mechanisms but is limited by light scattering and poor penetration in biological tissues. While tissue-clearing reagents (hydrophilic/hydrophobic) and bioluminescent probes improve imaging, achieving effective optical transparency in live tissues remains a challenge. This study builds on recent work using absorbing dyes (tartrazine and 4-aminoantipyrine) to enhance *in vivo* tissue clearing, aiming to optimize efficacy and biosafety.

**Aim:**

We aimed to develop a mixed solution of tartrazine and 4-aminoantipyrine (4-AA) that improves optical transparency, accelerates clearing, and reduces toxicity compared to individual dyes, enabling safer and more efficient deep-tissue imaging in live animals.

**Approach:**

The study employed a multi-pronged experimental approach: solution optimization involved testing varying ratios of tartrazine and 4-AA (5:1, 10:1) to characterize their optical properties through UV-Vis-NIR spectroscopy and refractive-index measurements, while simultaneously evaluating *ex vivo* skin-clearing efficacy; *in vivo* validation was conducted by applying the optimized gels to depilated mouse skin and systematically recording key parameters including transparency-onset time, maximum clearing duration, and light transmittance; concurrent biosafety assessments monitored critical health indicators such as animal survival rates, longitudinal weight changes, and liver/kidney function markers [alanine aminotransferase (ALT), aspartate aminotransferase (AST), and creatinine (CREA)] during the post-treatment period.

**Results:**

The optimized mixed solutions (5:1 and 10:1 tartrazine:4-AA ratios) demonstrated superior clearing efficiency, achieving faster tissue transparency than tartrazine alone while matching the performance of 4-AA but with significantly reduced toxicity. Optical characterization revealed stable refractive indices (∼1.42) and strong absorption across visible/NIR wavelengths for all formulations. While 4-AA alone exhibited severe hepatorenal toxicity and 100% mortality (3/3 mice), the 5:1 mixed solution maintained efficacy with no mortality and only mild ALT/AST elevation. Transmittance measurements showed 4-AA gels achieved ∼40% light transmission, whereas mixed gels reached ∼15% due to tartrazine’s residual absorption in the red–NIR spectrum, suggesting an optimal balance between clearing performance and biosafety in the composite formulations.

**Conclusions:**

The 5:1 tartrazine:4-AA cocktail optimally balances speed, clarity, and biosafety, advancing *in vivo* tissue-clearing technology. This strategy addresses key limitations of stand-alone dyes and expands potential applications in biomedical imaging, such as 3D tumor visualization and dynamic pathology studies. Future work should refine ratios for diverse tissues and integrate auxiliary agents (e.g., surfactants) to further enhance clearing.

## 1 Introduction

*In vivo* imaging technology holds profound significance in biomedical and fundamental scientific research, as it enables real-time acquisition of dynamic structural information from living organisms—a capability essential for applications such as real-time disease monitoring, disease diagnosis, pathology studies, and drug screening.^1^^,^^2^ However, *in vivo* imaging of live subjects is challenged by light scattering and absorption inherent to biological tissues. Composed of diverse substances such as water, lipids, and proteins, biological tissues exhibit substantial refractive index (RI) mismatches among these components, resulting in light scattering as light traverses the tissue, thereby markedly diminishing image resolution and contrast.^3^ Numerous previous studies have applied optical clearing agents to enhance light transmission and mitigate scattering, thereby improving imaging quality.^4^

Currently, tissue-clearing reagents can be broadly categorized into two types: hydrophilic and hydrophobic. Hydrophilic reagents have evolved from sucrose, fructose, and iodohexanol to more advanced formulations like antipyrine–nicotinamide (CUBIC) and N-methylacetamide–histodenz (Ce3D), all of which can achieve a high RI ranging from 1.33 to 1.56.^5^^,^^6^ Hydrophobic reagents are exemplified by benzyl alcohol–benzyl benzoate (BABB) and dibenzyl ether (DBE), which are commonly used for tissue clearing due to their ability to clear dense tissues effectively.^7^^,^^8^ Transparent tissue samples are typically imaged using techniques such as confocal microscopy, two-photon microscopy, light sheet microscopy, and optical projection tomography (OPT). Spinning disk confocal microscopy can capture 3D volume images but is limited by tissue depth. Two-photon microscopy extends the depth beyond the confocal limit but suffers from slower imaging speeds. Light sheet microscopy, which is often the preferred method for most transparent tissues, can achieve imaging depths of up to 100  μm;^9^ In comparison, OPT provides lower lateral resolution but higher axial resolution than light sheet or confocal microscopy using similar optical elements, and it can complete volumetric imaging in a matter of minutes to hours for appropriate samples.^10^

However, these conventional tissue-clearing techniques typically involve intricate preprocessing steps—including fixation, delipidation, staining, and refractive index matching—making them difficult to use for imaging in live subjects. In addition, these methods are not only time consuming but may also alter tissue structure and molecular properties.^11^^,^^12^ The highly toxic organic solvents, such as benzyl alcohol–benzyl benzoate (BABB), compromise tissue integrity. Consequently, while these techniques hold substantial promise for high-resolution microscopic imaging, their application in *in vivo* studies remains limited, particularly concerning biosafety and they may also cause fluorescence quenching, ultimately impacting imaging quality.^13^ In recent years, researchers have focused on identifying clearing agents and methodologies that are less toxic and more efficacious, aiming to achieve safe and efficient tissue clearing. These efforts are advancing the field of *in vivo* imaging technology, enhancing its applicability and potential within biomedical research.^14^^–^^16^

Besides these *ex vivo* tissue clearing methods, recent years have also witnessed the development of *in vivo* deep tissue imaging approaches. Optical imaging techniques using specially designed imaging probes, such as those with NIR-II fluorescence, bioluminescence, and chemiluminescence, have helped overcome the challenges of imaging deeper tissues.^17^^,^^18^ In addition, photoacoustic (PA) imaging represents another emergent technology that enables deep tissue imaging. Since the scattering of acoustic waves in biological tissues is much lower than that of photons, PA imaging provides high spatial resolution in deep tissues (≈6  cm).^19^ While these methods have made *in vivo* deep-tissue imaging possible, significant limitations remain in the observation of deeper tissues.

To address these challenges associated with *ex vivo* tissue clearing and *in vivo* deep tissue imaging, a recent paper introduced a novel approach to achieve *in vivo* optical transparency in live animals using food dyes such as tartrazine (also known as Yellow No. 5) and other absorbing molecules such as 4-aminoantipyrine (4-AA), as recently reported in *Science*.^20^ Their findings highlight the efficacy of these solutions in reducing light scattering and high biocompatibility, enabling longitudinal deep-tissue imaging in live mice.^21^ In this paper, we report the successful replication of the recent *Science* study and identify some limitations of this approach in its current stage. Specifically, while the tartrazine solution exhibits favorable biocompatibility for *in vivo* tissue transparency, it has a relatively low skin permeation efficiency, with its deep coloration potentially hindering imaging quality in live specimens. In contrast, another absorbing molecule identified in this paper, 4-AA, shows superior light transmission and skin permeation properties. However, we found that after treatment with a large amount of 38% 4-AA solution over prolonged periods, mice displayed significantly reduced vitality, with some experiencing mortality. Blood analysis revealed heightened hepatic and renal toxicity after prolonged treatment with 4-AA, suggesting potential biosafety concerns and limitations for long-term observation.

Building on the work of the recent *Science* study and our own experimental data, this report presents a new strategy using a mixed solution of tartrazine and 4-AA to yield improved imaging quality and higher efficiency in achieving transparency, while maintaining favorable biocompatibility, in live mice. This study aims to rationally design and optimize the ratio of tartrazine and 4-AA, balancing transparency efficacy with biosafety. We anticipate that this optimized formulation will offer a more efficient and safer solution for achieving *in vivo* tissue transparency, providing a promising pathway for the broader application of this technology in practical clinical settings.

## 2 Materials and Methods

### 2.1 Materials and Instruments

**Fig. 1:**
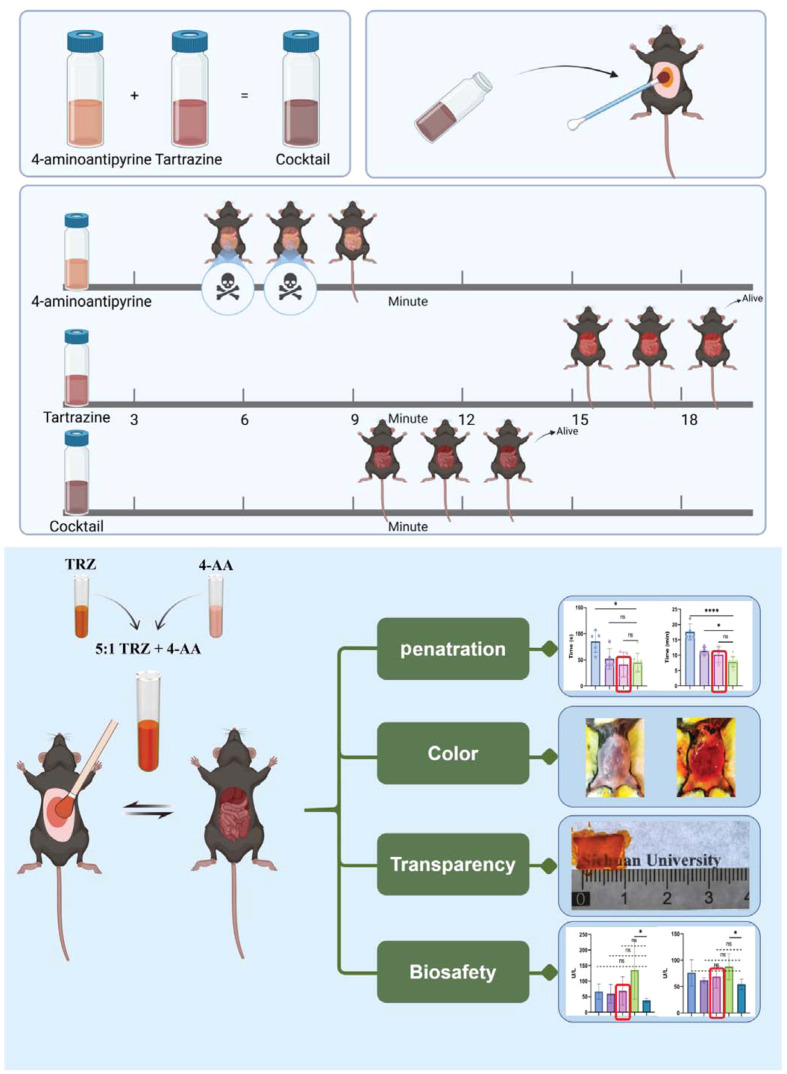
Schematic illustration of the experimental effects of different reagents. Compared with TRZ or 4-AA alone, 5:1 TRZ+4-AA mixed ratio reagent has the imaging efficiency and biological safety. TRZ: tartrazine, 4-AA: 4-aminoantipyrine.

In this experiment, by mixing absorbing molecules in varying proportions, a more optimized tissue-clearing effect can be achieved (Fig. 1). Experimental materials included the following: Tartrazine (T0388, industrial grade, purity ≥85%) and 4-AA (A4382, analytical grade) were procured from Sigma Aldrich. Low-melting-point agarose (A9414, CAS: 39346-81-1) was also obtained from Sigma Aldrich. Experimental animals included female C57BL/6 mice and Sprague-Dawley (SD) rats, sourced from Beijing HFK Bioscience Co., Ltd. (Beijing, China). The animals were housed under specific pathogen-free (SPF) conditions, and all experimental procedures strictly adhered to ethical and welfare guidelines for animal research at West China Hospital and Sichuan University (Ethics No.20241202008).

Instruments used in this study included a UV-Vis-NIR spectrophotometer (LAMBDA 1050+, PerkinElmer), an Abbe refractometer (Shanghai Lichen Instrument Technology Co., Ltd.), a thermostatic water bath (Thermo Fisher), a precision balance (METTLER TOLEDO ME204), an optical microscope (Nikon Eclipse Ni-U), dissection tools (scalpels, tweezers, and dissection boards), a custom-modified uniform white lightbox, imaging equipment (Canon EOS 90D), a biosafety cabinet (ESCO Class II), a hematology analyzer (Sysmex XN series), and a −80°C freezer (Haier DW-86L626).

All procedures were conducted in clean laboratories and animal facilities, maintained at a temperature of 22±2°C and a relative humidity of 50%±10%.

### 2.2 Replication of the Original Study

The preparation of dye solutions and gels followed the published protocol in the original *Science* paper.^20^ Specifically, for dye solutions, tartrazine and 4-AA were weighed and placed into a 20 mL reagent vial, according to the ratios listed in Table S1 in the Supplementary Material. A corresponding amount of distilled water was added to produce a total mass of the mixture of 5 g, and the mixture was thoroughly vortexed. The vial was then incubated in a preheated water bath or oven at 70°C to 80°C for 10 min to ensure complete dissolution of both absorbing molecules [Fig. 3(a)]. Once fully dissolved, the solution was ready for use in immersion experiments involving the dissected rat skin. Skin samples were excised from the dorsal region of SD rats and sectioned into approximately 1×1  cm squares before use. The *ex vivo* tissue samples were immersed in the solution of the corresponding absorbing molecules for 24 h, after which their transparency was assessed on the lightbox.

For *in vivo* experiments, 30 mg of low-melting-point agarose was added to the aforementioned solution, which was again placed in the 70°C to 80°C water bath for 10 min until the agarose completely dissolved. The vial was gently shaken to ensure uniform distribution, resulting in a homogeneous and transparent suspension. The suspension could be topically applied either as a liquid or allowed to gradually form a gel upon cooling to room temperature. Both the liquid and the agarose-containing gel were suitable for *in vivo* applications.

Five C57BL/6 mice, aged 3 to 4 weeks and weighing 8 to 15 g, were selected for the *in vivo* experiment. Mice were anesthetized using isoflurane inhalation following the APLAC protocol in our university. The abdominal area was treated with depilatory cream, left for 5 min, and then wiped clean with alcohol swabs to remove hair and residual cream. This step was repeated to eliminate hair roots and gently dissolve the epidermis, while minimizing the risk of skin burns.

The gel containing absorbing molecules was evenly applied to the depilated skin surface using a cotton swab. Gentle massage was performed for 1 to 3 min while observing and recording the time taken for the skin to develop an initial orange-red transparent window. Massage was continued for an additional 5 to 10 min to achieve maximum transparency, and the required time was also recorded. At the end of each experiment, the mouse abdomen was wiped with saline to remove the gel and extract any residual absorbing molecules in the skin, restoring the skin to its original opaque state.

### 2.3 Preparation of Cocktails and Optical Characterizations

#### 2.3.1 Solution preparation

Following the ratios specified in Table S1 in the Supplementary Material, solutions were prepared with tartrazine and 4-AA at weight ratios of 10:2.53 and 10:1.27, which correspond an equivalent ratio of 5:1 and 10:1 for these two molecules as defined in the *Science* paper, respectively. Low-melting-point agarose was not added to these solutions.

#### 2.3.2 Optical characterizations

The transmission spectra of the solutions were measured using a UV-Vis-NIR spectrophotometer (LAMBDA 1050+, PerkinElmer). The instrument was set to measure wavelengths ranging from 200 to 1000 nm, with a scanning speed of 300 nm/min. A 10 mm optical-path quartz cuvette (PerkinElmer) was used. Preparation steps included rinsing the cuvette with pure water, drying it, and then acquiring a reference spectrum using only pure water to correct for inherent optical losses caused by absorption or reflection at the air–quartz interface before carrying out the measurements for specific solutions.

The refractive indices of the solutions were measured using an Abbe refractometer (Shanghai LICHEN INSTRUMENT Technology Co., LTD). The refractometer was placed in a well-lit area, calibrated with a standard liquid of known refractive index prior to measurements, and cleaned with anhydrous ethanol to ensure the prisms were free from residues. A few drops of the sample solution were placed on the rough surface of the auxiliary prism using a pipette, and the auxiliary prism was quickly closed. After adjusting the eyepiece, the refractive index was recorded from the scale through the reading telescope. Each solution’s refractive index was measured three times, and the average value was calculated.

To visually assess transparency, *ex vivo* rat skin samples were immersed in these solutions for 24 h. Changes in skin transparency were observed by placing the samples over a sheet of paper printed with the text “Sichuan University.” The clarity of the text through the skin was photographed and documented.

### 2.4 Evaluation of Clearing Performance *In Vivo*

Different gel formulations were applied to the abdominal skin of mice for achieving optical transparency (n=5 to 8), following the method described in Sec. 2.2. The time at which the first appearance of the orange-red transparent window was observed was recorded, as well as the time required to achieve maximum transparency. Statistical analysis was conducted on both time points to compare the transparency efficiency of gels with different ratios of tartrazine and 4-AA.

After optical transparency was achieved, mice were euthanized and the abdominal skin was dissected immediately for quantitative transmittance measurements. Specifically, transmittance was measured using a spectrophotometer to assess the transparency effect of different solutions, normalized against a control skin sample dissected from an untreated and still opaque skin region of the same mouse abdomen.

### 2.5 Evaluation of Long-term Biosafety of Different Absorbing Molecule Formulations

Grouping and solution treatment: Experimental mice were randomly assigned into five groups, each receiving a different formulation of absorbing molecule solution (e.g., 5:1 tartrazine:4-AA, 10:1 tartrazine:4-AA, tartrazine alone, 4-AA alone, and a control group with saline alone). Sample sizes were selected to ensure statistical validity. Mice were subjected to skin transparency treatments, recovered, and subsequently monitored at predetermined time intervals according to the experimental design.

Blood analysis: Blood samples were collected on Day 1 and Day 3 post-treatment for analysis of liver function markers [alanine aminotransferase (ALT); aspartate aminotransferase (AST), and renal function markers (serum creatinine, CREA)].

Weight and survival monitoring: Survival rates and body weight changes were recorded from the start of the experiment until Day 14 post-treatment. Growth trends of body weights were monitored, and any abnormal weight loss or signs of distress were noted to assess the potential long-term toxicity of the solutions.

## 3 Results and Discussion

### 3.1 Study Design and Initial Observations

**Fig. 2:**
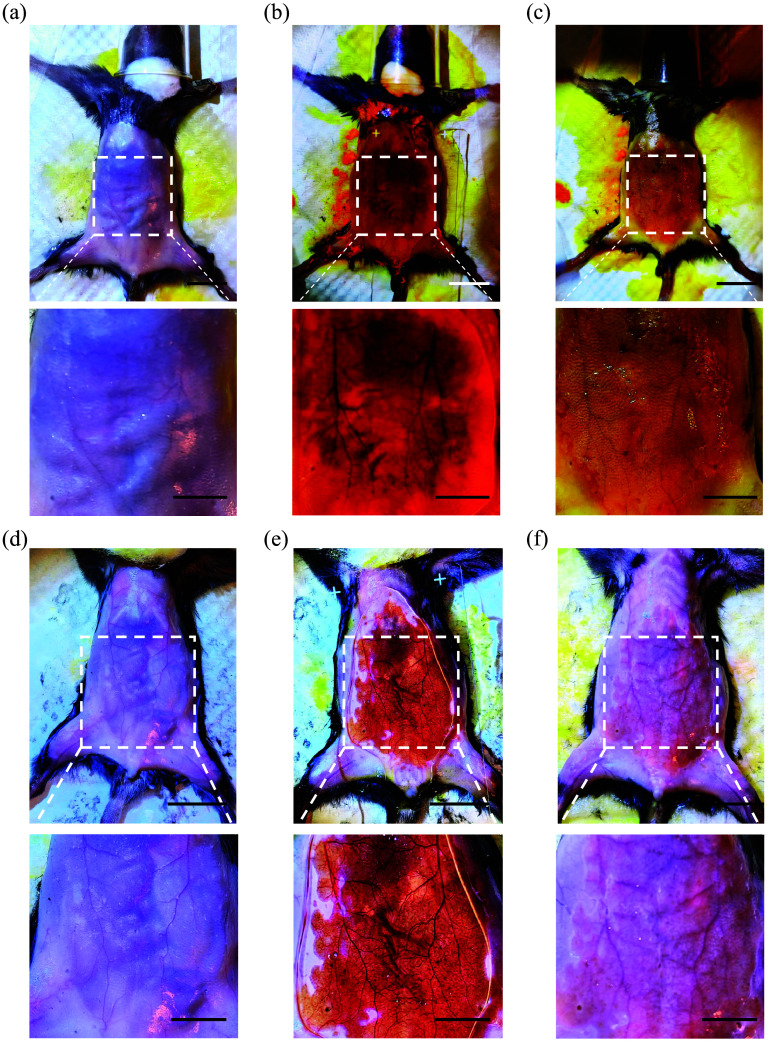
(a)–(c) Brightfield images of mouse abdomen before, during, and after the clearing process using 30% tartrazine; (d)–(f) Brightfield images of mouse abdomen before, during, and after the clearing treatment with 38% 4-AA. Scale bars, 10 mm; Scale bars of zoom-in image, 5 mm.

We prepared a live animal clearing solution by refining the methodology in the original publication^20^ and successfully reproduced the clearing effect reported in the original study [Figs. 2(a)–2(f) and Figs. S1 and S2 in the Supplementary Material]. This confirmed the effectiveness of tartrazine solution and 4-AA as live animal clearing agents. In addition, we applied this solution to the spine, achieving clearing effects not reported in the original study (Fig. S3 in the Supplementary Material), highlighting the promising potential for broader applications of this approach in the future.

However, during the experiments, we identified several challenges that may impact the further development of this approach. First, the clearing rate of tartrazine solution is relatively slow, requiring more time to massage and penetrate the skin, which prolongs both the experimental duration and anesthesia time. In addition, due to its dark orange-to-red color, the final imaging results are often affected by the resulting color of the skin. On the other hand, while 4-AA demonstrated a faster penetration rate and better clearing effects, we observed high mortality among the mice initially treated with 4-AA (3/3). Previous studies have established that 4-AA is an active metabolite of analgin with demonstrated anti-inflammatory and sedative properties.^22^^,^^23^ As a precaution, we reduced the depth of anesthesia, yet mortality remained high (2/5). After conducting blood analysis on the deceased mice, we found that their liver and kidney functions were severely affected. Furthermore, we discovered that the oral lethal dose (LD50) of 4-AA is one-fifteenth of that of tartrazine (LD50: 800  mg/kg for 4-AA^24^ versus >12750  mg/kg for tartrazine^25^). In our experiments, we estimated that approximately 300 mg of 4-AA was applied to the skin of each mouse, a dosage that significantly exceeds the recommended safety limit. After further consideration, we decided to combine the two solutions of tartrazine and 4-AA and optimize the depth of anesthesia. We first prepared a 1:1 mixture of tartrazine and 4-AA. When applied to mice, the penetration rate improved significantly, and the imaging effect was satisfactory. However, a small number of mice still died (1/5), suggesting potential toxic effects of 4-AA. To investigate this, we collected serum samples from mice treated with three different clearing solutions and observed a noticeable yellow discoloration in the serum of tartrazine mice. Subsequent testing of relevant indicators (Table S2 in the Supplementary Material) revealed that the concentration of 4-AA directly affects liver and kidney function once absorbed into the bloodstream. These findings highlight the need to further optimize the solution ratio and rigorously evaluate its safety.

### 3.2 Spectral Characterizations of Solutions in Varying Ratios and *Ex Vivo* Experimental Analysis

**Fig. 3:**
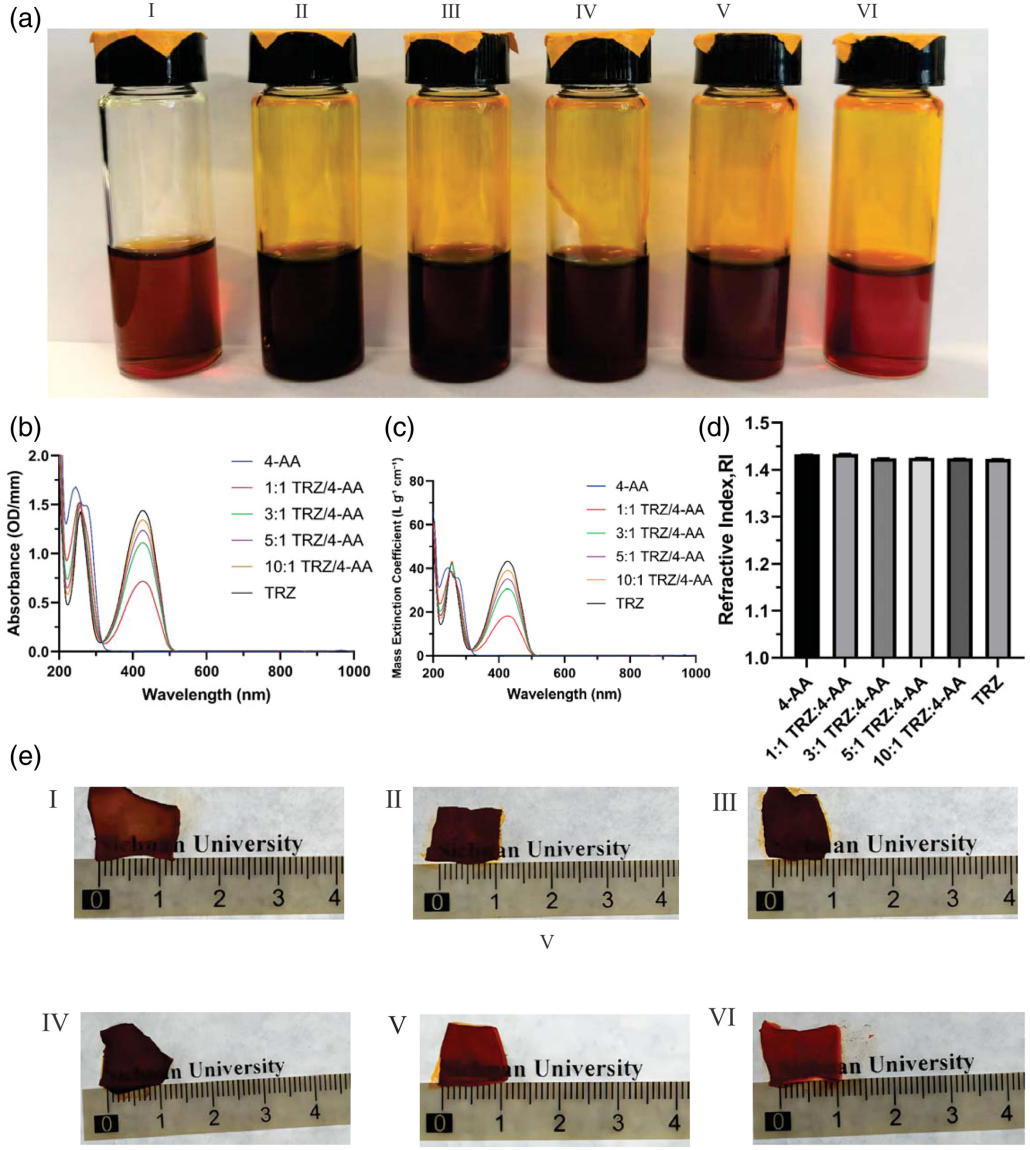
(a) Gels with different ratios of tartrazine to 4-AA. I–VI represent the following compositions: 4-AA alone, 1:1 tartrazine:4-AA, 3:1 tartrazine:4-AA, 5:1 tartrazine:4-AA, 10:1 tartrazine:4-AA, and tartrazine alone; (b) Absorption spectra of the solutions at different ratios in the wavelength range of 200 to 1000 nm. Spectra were recorded after a 10,000-fold dilution, while the plotted molar extinction coefficients were calculated based on the undiluted concentrations; (c) The mass extinction coefficient spectra of each solution, calculated according to ε=A/(c×l) based on the measured absorption spectra and the volume of the prepared solutions; (d) Refractive indices (RI) of different solutions. Data are represented as mean ± standard deviation (SD); (e) Brightfield images of rat skin soaked in different solutions. The thickness of rat skin is approximately 2 mm. I–VI represent the following compositions: 4-AA alone, 1:1 tartrazine:4-AA, 3:1 tartrazine:4-AA, 5:1 tartrazine:4-AA, 10:1 tartrazine:4-AA, and tartrazine alone. TRZ: tartrazine, 4-AA:4-aminoantipyrine.

To further optimize the solution’s effectiveness, we prepared cocktails with various ratios [Fig. 3(a)] and measured their UV-visible absorbance spectra [Fig. 3(b)] and RI [Fig. 3(d)]. The mass extinction coefficient spectra of each solution were then calculated according to ε=A/(c×l) based on these measured absorption spectra and the prepared solution volumes. The intense absorption profiles observed in the UV-visible absorbance spectra and the relatively consistent results of the RI suggest that tartrazine and 4-AA do not chemically react with each other and can be dissolved in any ratio to form a stable tissue-clearing solution with the same RI of approximately 1.42. Based on these spectral characterization data as well as animal experiments above, we inferred that a tartrazine to 4-AA ratio of 5:1 to 10:1 may yield improved results. To test this hypothesis, we applied these new cocktail solutions to *ex vivo* rat skin and achieved promising clearing effects [Fig. 3(e)].

### 3.3 *In Vivo* Experimental Analysis and Evaluation of Clearing Efficacy

**Fig. 4:**
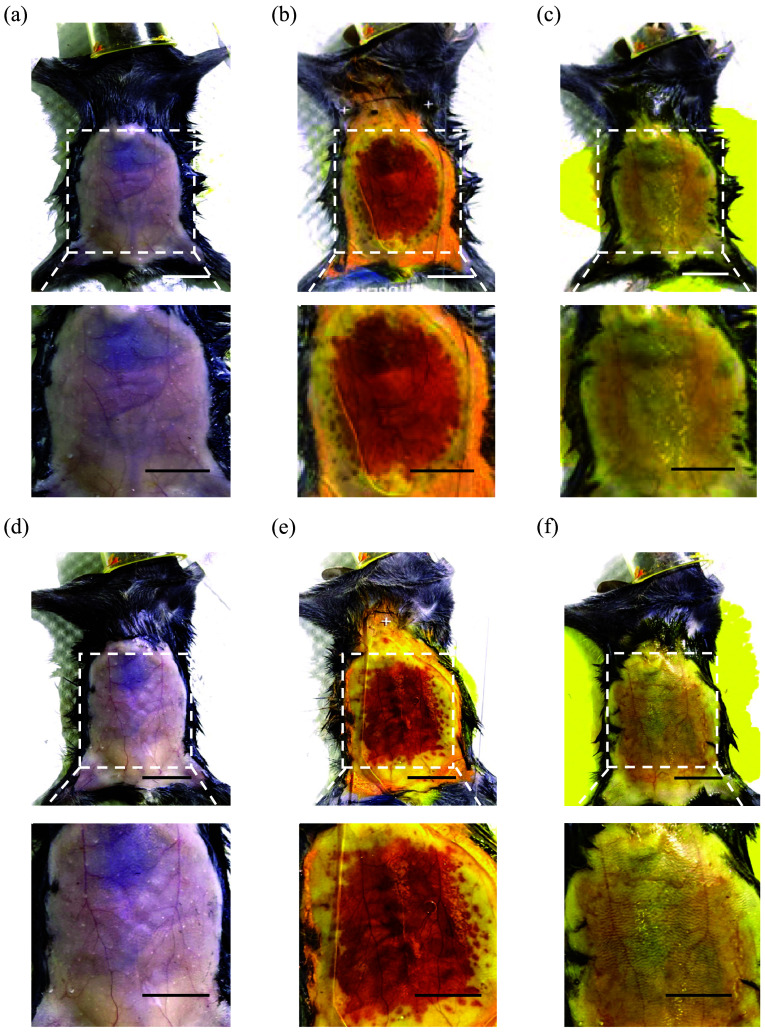
(a)–(c) Brightfield images of mouse abdomen before (a), during (b), and after (c) the clearing treatment with 5:1 tartrazine:4-AA, respectively; (d)–(f) Brightfield images of mouse abdomen before (d), during (e), and after (f) the clearing treatment with 10:1 tartrazine:4-AA, respectively. 4-AA: 4-aminoantipyrine. Scale bars, 10 mm; Scale bars of zoom-in image, 5 mm.

**Fig. 5:**
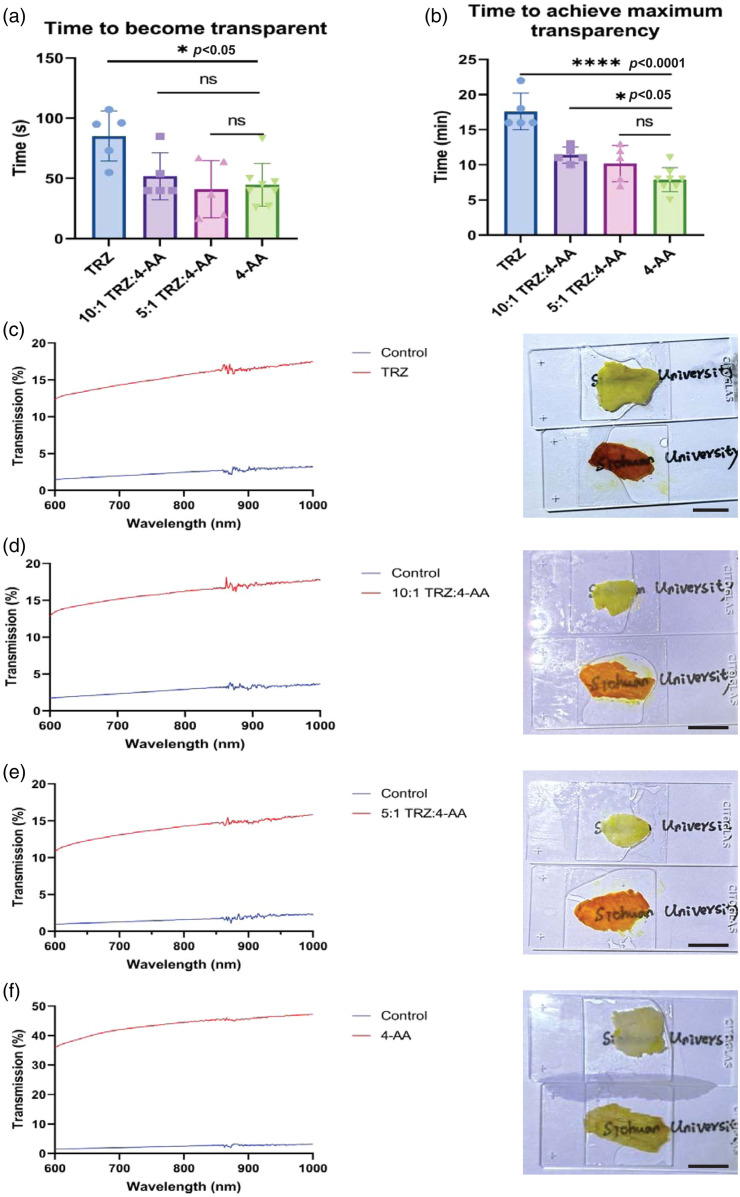
(a) Time to initial appearance of transparent window in different groups; (b) Time to maximum transparency in different groups. TRZ: tartrazine, 4-AA: 4-aminoantipyrine. Data are represented as mean ± standard deviation (SD). *: p<0.05; ****: p<0.0001; ns: not significant; (c)– (f) Transmission spectra of mouse abdominal skin after treatment with gels of different compositions. The image on the right of each panel shows the comparison between the skin without gel treatment and the skin treated with gel. In each image, both skin samples were obtained from the abdomen of the same mouse. TRZ: tartrazine, 4-AA: 4-aminoantipyrine. Scale bars, 10 mm.

We then applied gels with various ratios of tartrazine and 4-AA to make mouse abdominal skin transparent (n=5 to 8, Fig. 4). We observed that while the tartrazine percentage was reduced, the clearing effect improved, with a caution that the clearing effect could be influenced by the skin color of the mice. We recorded the time at which the orange-red transparent window first appeared and the time when the maximum clearing effect was achieved. The results showed that both the 5:1 and 10:1 gels produced orange-red transparent windows relatively quickly. There was no significant difference between the time taken for the 5:1 and 10:1 gels and the 4-AA gel to produce the initial transparent window [Fig. 5(a)], nor was there a statistical difference between the time taken by the 5:1 gel to achieve the maximum clearing effect compared to the 4-AA gel [Fig. 5(b)].

To further quantify the clearing effect, we measured the light transmittance of mouse skin treated with these dye-containing gels [Figs. 5(c)–5(f)]. Skin treated with the 4-AA gel achieved a light transmittance of approximately 40%, while the other three gels reached around a transmittance of 15%. The lower transmission of the latter three gels was attributed to the presence of tartrazine, which has significant lingering absorption in the red to near-infrared region of the spectrum.

### 3.4 Evaluation of Biosafety

**Fig. 6:**
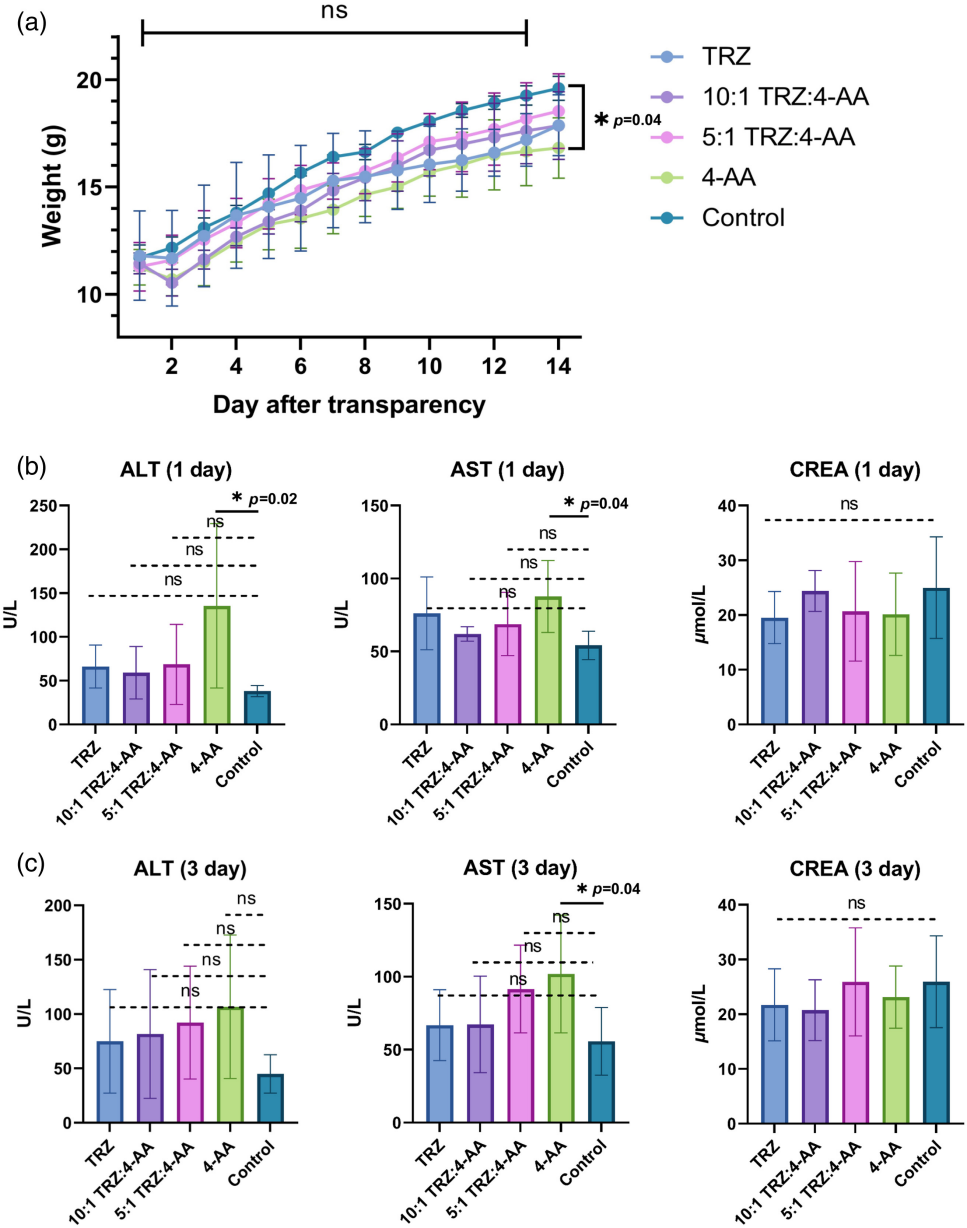
(a) Body weight changes of mice treated with different gels and those in the control group without clearing treatment. No statistical difference was found in body weight between any groups on the first 13 days after treatment, while a statistical difference was found in body weight between control group and 4-AA group on Day 14 after treatment; (b) and (c). Changes in ALT, AST, and CREA levels in mice treated with different gels and those in the control group without clearing treatment on Day 1 and Day 3 after treatment. TRZ: tartrazine, 4-AA: 4-aminoantipyrine; ALT: Alanine aminotransferase; AST: Aspartate aminotransferase; CREA: serum creatinine; *: p<0.05; ns: not significant.

Liver and kidney toxicity represents the most critical biosafety concern for each solution used in our approach. As a result, we monitored body weight changes [Fig. 6(a)] and blood indicators [Figs. 6(b) and 6(c)] in mice treated with gels of different ratios of tartrazine and 4-AA. The results showed that the 5:1 and 10:1 gels were more effective in minimizing liver and kidney toxicity. However, the differences in liver and kidney function among these five groups of mice were not statistically significant. We speculate that this phenomenon may be related to individual variability in the mice’s tolerance to 4-AA, as mice with poorer tolerance might have died during the experiment. While the mice experienced a slight weight decrease on the first day following gel application (with tartrazine showing the least decrease), their weight steadily increased thereafter, and all mice survived. These findings suggest that both 5:1 and 10:1 gels are within a safe range for use in live animals.

### 3.5 Comparative Discussion and Potential Applications

In this study, we demonstrated that both tartrazine and 4-AA individually exhibit excellent tissue-clearing effects. Additionally, the mixed solution of tartrazine and 4-AA produced comparable results, particularly in clearing both living and *ex vivo* tissues. Notably, the 5:1 ratio of tartrazine to 4-AA achieved the optimal balance of clearing effectiveness and safety.

Tartrazine, a yellow water-soluble anionic azo dye, is widely used in food, cosmetics, and biological research. As a result, it has been extensively studied in various laboratories to assess its potential genotoxicity,^26^ cytotoxicity,^27^ carcinogenicity,^28^ developmental toxicity,^29^ as well as its potential neurological, reproductive, and endocrine effects. Several studies have reported that tartrazine induces various blood biochemical changes, including alterations in liver enzymes, creatinine, uric acid, and lipids. In severe cases, these changes may lead to pathological damage in the liver and kidneys.^30^^,^^31^ In our experiments, no deaths occurred in mice treated with tartrazine. When we observed mortality in 4-AA–treated mice during the exploratory experiments, we also assessed the liver and kidney functions in tartrazine-treated mice. Although tartrazine had some impact on the organ functions of individual mice, its severity was significantly lower than that of 4-AA. This difference may be attributed to the oxidative stress induced by tartrazine in multiple organs.^32^ When tartrazine was used for *in vivo* imaging in mice, its darker color resulted in suboptimal transparency visible to the naked eye, but it had fewer side effects.

4-AA is the active metabolite of the analgesic drug analgin, which is still used in some developing countries. It shares similar anti-inflammatory and analgesic effects as the parent drug, functioning by inhibiting the synthesis of prostaglandins through the inhibition of cyclooxygenase (COX). This action blocks pain signal transduction, providing relief from pain and fever.^33^ Studies have shown that 4-AA can mitigate the effects of chemotherapy drugs that induce DNA damage, helping to prevent gene and chromatin damage.^34^ However, at the outset of our study, all mice treated with 4-AA for tissue clearing died. Upon testing, we found that the liver and kidney functions of these mice were worse than those treated with tartrazine. In contrast, the liver and kidney functions of mice treated with the 1:1 mixed solution were intermediate between those of the tartrazine and 4-AA groups.

The combined use of these two compounds at rationally designed ratios significantly enhances the transparency of the tissue, providing higher-quality specimens for biological imaging, particularly for three-dimensional imaging techniques such as light sheet microscopy and confocal microscopy. Additionally, this combination helps mitigate the impact on liver and kidney function in living animals, as indicated in Fig. 6. Transparent samples offer clear visualization of local tissue structures and cell relationships, which is valuable for investigating biological processes such as cell dynamics, cancer metastasis, and nervous system development. In contrast to traditional medical imaging techniques like MRI, CT, and X-ray, which typically provide only two-dimensional images and have limitations in imaging fine tissues, living tissue transparency technology enables the volumetric imaging and three-dimensional reconstruction of biological tissues or organs. This provides more detailed imaging information. For instance, using transparency technology, doctors can observe the size, shape, and spatial relationship of tumors with surrounding tissues in three dimensions, enabling more accurate diagnoses and treatment plans. In addition, transparent tissues can be further analyzed using advanced imaging technologies, such as PET scans, which enhance the detection accuracy and precision of diseased tissues, aiding in the early diagnosis of diseases. In pathological research, transparency technology offers a new approach to observing tissue sections. Traditional pathological sections, when examined through an optical microscope, are often limited by factors such as slice thickness and light scattering. However, samples processed with transparency technology provide a clearer, three-dimensional view of tissue structure, improving pathologists’ ability to identify disease processes, tissue changes, and affected areas. This advancement holds significant value for the early diagnosis and targeted treatment of cancers, immune diseases, degenerative disorders, and more.

Although the mixed solution of tartrazine and 4-AA demonstrates significant tissue-clearing effects, different tissues and organs may respond differently to clearing agents. Therefore, further optimizing the reagent ratio to suit the clearing needs of various tissue types and organs is a key area for future research. For instance, adjusting the concentrations of tartrazine and 4-AA or incorporating additional components, such as active surfactants as solubilizers or organic solvents, could enhance the clearing effect and shorten the imaging window.

In summary, the cocktail solutions of tartrazine and 4-AA show great potential in tissue-clearing technology, particularly in medical and biological imaging. This study not only enhances the clearing effect but also offers a new direction for technological innovation and application in related fields. However, further research is needed to optimize the reagents, assess safety, and explore new materials to ensure the broader adoption and evaluation of this technology in clinical and research settings.

## Supplementary Material

10.1117/1.JBO.31.5.054702.s01

## Data Availability

All data in support of the findings of this paper are available within the article or as Supplementary Material.
