# Supplementary material for: Enhancing the efficiency of achieving optical transparency in live animals using absorbing molecules
Source: J Biomed Opt. 2026 Feb 13;31(5):054702. doi: 10.1117/1.JBO.31.5.054702 (PMC12904770; doi:10.1117/1.JBO.31.5.054702)
Supplement: Supplementary file 1 [file JBO_031_054702_SD001.pdf]

Supplementary Table 1. The composition ratio of different proportions of solution or gel.

	4-AA (g)	Tartrazine (g)	ddH_2O (ml)	Agarose (mg)
30 w/w % tartrazine sodium salt	0	1.5	3.5	30
10:1 tartrazine:4-AA	0.173	1.364	3.463	30
5:1 tartrazine:4-AA	0.316	1.25	3.434	30
3:1 tartrazine:4-AA	0.475	1.125	3.4	30
1:1 tartrazine:4-AA	0.95	0.75	3.3	30
38 w/w % 4-AA	1.9	0	3.1	30

Supplementary Table 2. The liver and kidney functions of the three mice in the exploratory experiment.

	ALT (U/L)	AST (U/L)	CREA (μmol/L)
TRZ	785.25	762.22	28.35
1:1 tartrazine:4-AA	749.29	637.77	50.12
4-AA	1019.16	752.08	80.09

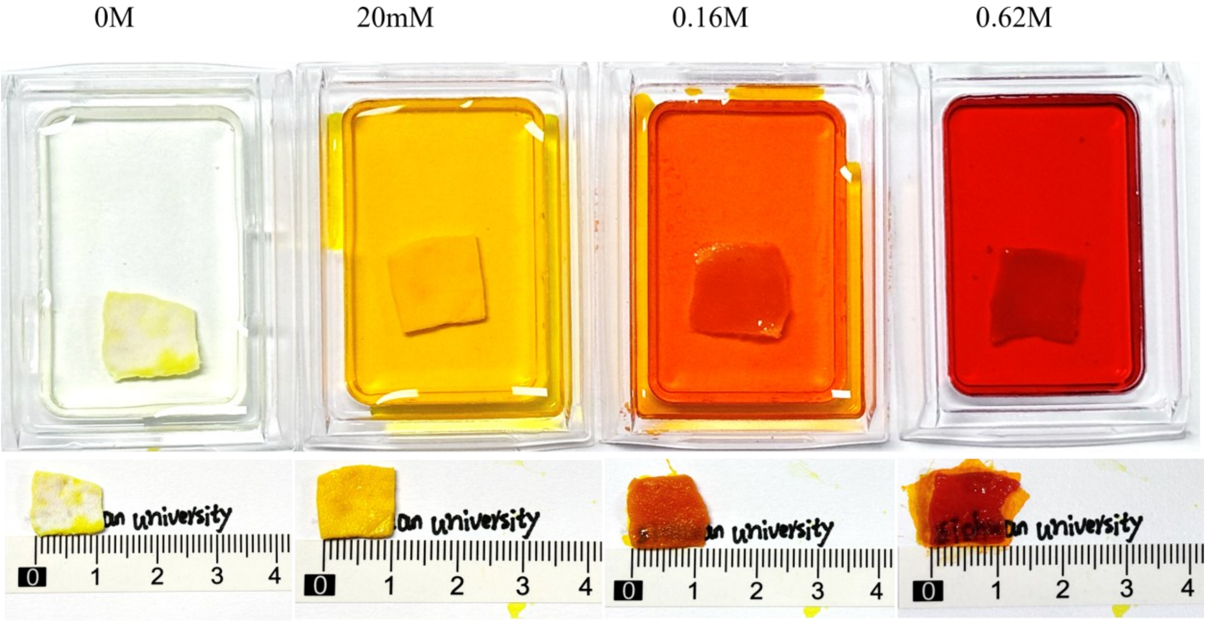

Supplementary Figure 1. Rat skin soaked in 0 M, 20 mM, 163 mM, and 0.62 M concentrations of tartrazine. The thickness of rat skin is approximately 2 mm.

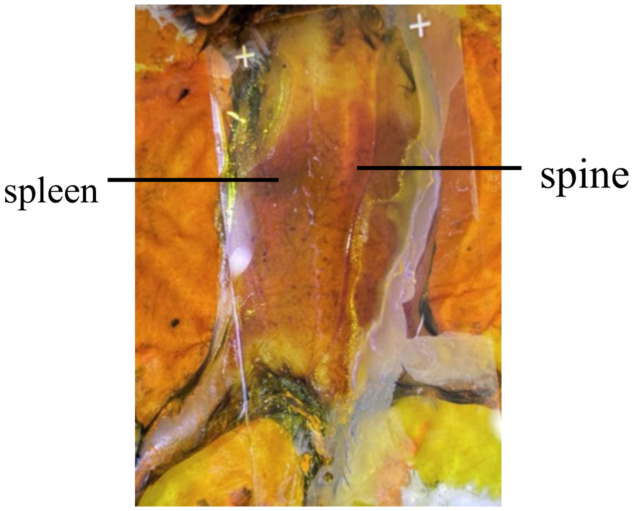

Supplementary Figure 2. Images of the mouse back treated with 30% tartrazine for clearing, showing a clear spinal column.

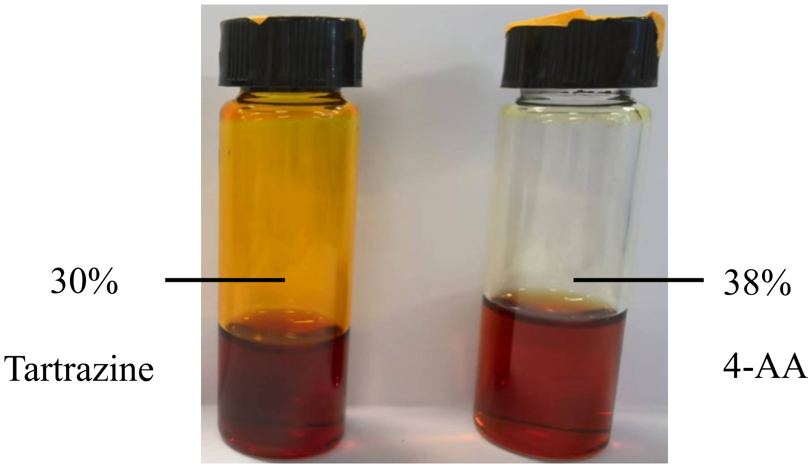

Supplementary Figure 3. Images showing the vials containing the two types of gels.

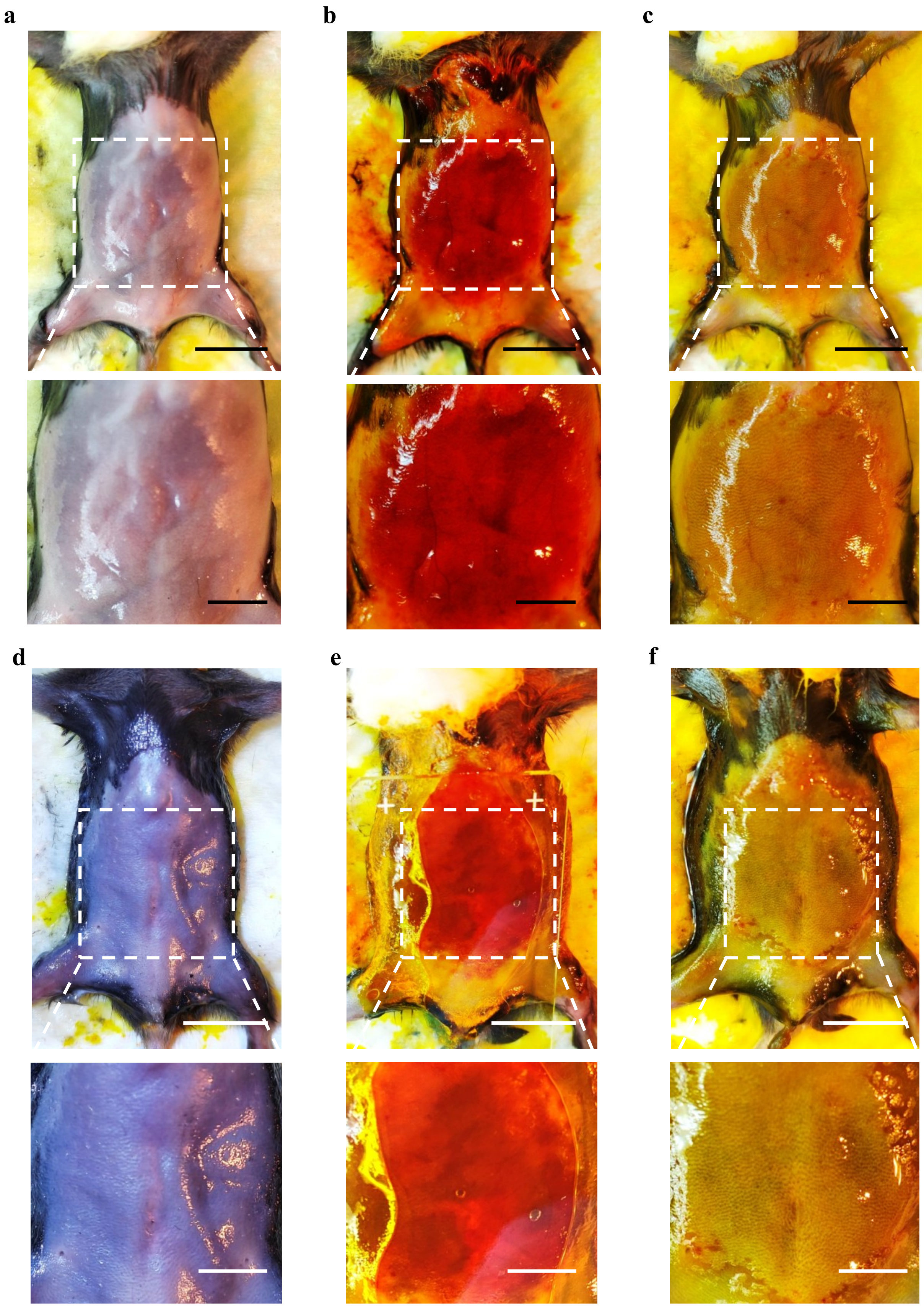

Supplementary Figure 4. (a)–(c) Brightfield images of mouse abdomen before(a), during (b), and after (c) the clearing treatment with 5:1 tartrazine:4-AA, respectively; (d)–(f) Brightfield images of mouse abdomen before (d), during (e), and after (f) the clearing treatment with 10:1 tartrazine:4-AA, respectively. 4-AA: 4-aminoantipyrine. Scale bars, 10 mm; Scale bars of zoom-in image, 5 mm.
